# Intrarectal quinine for treating *Plasmodium falciparum *malaria: a systematic review

**DOI:** 10.1186/1475-2875-4-24

**Published:** 2005-05-18

**Authors:** Michael Eisenhut, Aika Omari, Harriet G MacLehose

**Affiliations:** 1 Arrowe Park Hospital, Wirral Hospital NHS Trust, Upton, Wirral, UK; 2 Paediatric Department, Glan Clwyd Hospital, Clwyd, UK; 3 International Health Research Group, Liverpool School of Tropical Medicine, Liverpool, UK

## Abstract

**Background:**

In children with malaria caused by *Plasmodium falciparum*, quinine administered rectally may be easier to use and less painful than intramuscular or intravenous administration. The objective of this review was to compare the effectiveness of intrarectal with intravenous or intramuscular quinine for treating falciparum malaria.

**Methods:**

All randomized and quasi-randomized controlled trials comparing intrarectal with intramuscular or intravenous quinine for treating people with falciparum malaria located through the following sources were included: Cochrane Infectious Diseases Group Specialized Register, CENTRAL, MEDLINE, EMBASE, LILACS and CINAHL. Trial quality was assessed and data, including adverse event data, were extracted. Dichotomous data were analysed using odds ratios and continuous data using weighted mean difference.

**Results:**

Eight randomized controlled trials (1,247 children) fulfilled the inclusion criteria. The same principal investigator led seven of the trials. Five compared intrarectal with intravenous quinine, and six compared intrarectal with intramuscular treatment. No statistically significant difference was detected for death, parasite clearance by 48 hours and seven days, parasite and fever clearance time, coma recovery time, duration of hospitalization and time before drinking began. One trial (898 children) reported that intrarectal was less painful than intramuscular administration.

**Conclusion:**

No difference in the effect on parasites and clinical illness was detected for the use of intrarectal quinine compared with other routes, but most trials were small. Pain during application may be less with intrarectal quinine. Further larger trials, in patients with severe malaria and in adults, are required before the intrarectal route could be recommended.

## Background

*Plasmodium falciparum *malaria often causes serious illness, particularly in Africa, South-East Asia and South America. An estimated 200 million episodes of clinical malaria and two million deaths occur in children under five years old in Africa every year [1].

Uncomplicated malarial illness is usually treated with oral drugs [2]. But as vomiting is a prominent feature in 30 to 50 per cent of people with *P. falciparum *malaria [3-6], those who present to hospital with persistent vomiting (regardless of severity of disease) or severe malaria require other routes of administration, such as intravenous infusion, intramuscular injection [7] or via the nasogastric route. These different routes of administration require trained staff and equipment, which may be in short supply in low- and middle-income countries.

Despite emerging resistance to commonly used drugs, such as chloroquine and mefloquine, malaria parasites remain sensitive to quinine in Africa [8,9]. In some parts of South-East Asia, however, decreasing sensitivity to quinine has been detected [10].

Although intramuscular injection is the most common route of quinine administration used in low-and middle-income countries, side effects have been reported [11]. In some of these countries, it is the most common cause of lower limb paralysis when administered mistakenly into the sciatic nerve [12-14]. Other reported harmful effects of intramuscular quinine injections are bacterial and viral infections including tetanus [15], poliomyelitis [16,17] and HIV [18,19]. An alternative to intravenous and intramuscular administration is, therefore, worth evaluating.

The intrarectal route has been used to give quinine [20]. Health workers with minimal training can give intrarectal quinine to people who are either vomiting or comatose. This provides early treatment of the illness and is one of the strategies of the World Health Organization initiative 'Roll Back Malaria' . However, disadvantages of using the intrarectal route are local irritation, diarrhoea and expulsion of the medication [21]. The likelihood of intrarectal irritation has been reduced by the development of less acidic quinine gluconate (in Quinimax^®^). People may also reject suppositories and other intrarectal formulations in preference for the intramuscular route because injections are perceived as a more effective treatment, particularly in people who are seriously ill [19].

This review summarizes existing trials that compare the effectiveness and safety of intrarectal quinine with other routes of administration in people with malaria caused by *P. falciparum*.

The paper is based on a Cochrane review published in *The Cochrane Library *2005, Issue 1 [22].

Cochrane reviews are regularly updated as new evidence emerges and in response to comments and criticisms, and *The Cochrane Library *should be consulted for the most recent version of the review. The conclusions presented are the opinions of review authors and are not necessarily shared by The Cochrane Collaboration.

## Methods

The review's inclusion criteria were randomized and quasi-randomized controlled trials comparing intrarectal with intravenous or intramuscular quinine administration in patients with *P. falciparum *malaria (uncomplicated or severe, and confirmed by a blood-slide examination). Quinine could be used as a single therapy or in combination.

The review's primary outcome measure was death, but parasite clearance by 48 hours (number of participants free of parasites by 48 hours), parasite clearance by day seven (number of participants free of parasites by day seven), parasite clearance time, fever clearance time, duration of hospitalization, coma recovery time, time lapse before drinking or eating, and adverse events (serious events that resulted in death, were life-threatening, required hospitalization or resulted in discontinuation of treatment, such as local pain, abscess formation, and paralysis, mild or moderate adverse events, as classified or defined by trial investigators, including vertigo and tinnitus) were also measured.

The search strategy aimed to identify all relevant trials regardless of language or publication status (published, unpublished, in press and in progress). The following search terms were used: 'quinine', 'Quinimax^®^', 'cinchona alkaloids', 'cinchona alkaloid', 'suppositories', 'suppository', 'rectal drug administration', 'administration, rectal', 'intrarectal', 'rectal', 'rectum' and 'malaria'. The following databases were searched: Cochrane Infectious Diseases Group Specialized Register (January 2005), Cochrane Central Register of Controlled Trials (CENTRAL) published in *The Cochrane Library *(Issue 4, 2004), MEDLINE (1966 to January 2005), EMBASE (1974 to January 2005), LILACS (1982 to January 2005) and CINAHL (1982 to January 2005). The following conference proceedings were also searched for relevant abstracts: The Third Multilateral Initiative on Malaria Pan-African Conference, 18 to 22 November, 2002, Arusha, Tanzania, and the Third European Congress on Tropical Medicine and International Health held in Lisbon, Portugal (September, 2002). For unpublished or ongoing trials, individual researchers working in the field and the pharmaceutical company Sanofi-Synthélabo, which manufactured Quinimax^® ^suppositories and intrarectal cream, were contacted. The reference lists of all studies identified by the above methods were also checked.

The full reports for all potentially relevant trials were retrieved and independently assessed for their eligibility. For methodological quality, allocation sequence and allocation concealment were independently assessed to be adequate, inadequate or unclear according to established guidelines [23]. It was noted whether the participant, carer or outcome assessor was blind to the intervention, and the inclusion of all randomized participants in the final analysis was considered to be adequate if greater than 90%. Data were independently extracted using standard forms, and trial authors were contacted where additional unpublished data were required. Data were analysed using Review Manager 4.2 software and outcome measures were compared using odds ratios (OR) for dichotomous data and weighted mean difference (WMD) for continuous data, both with 95% confidence intervals. The fixed-effect model was used for those without statistically significant heterogeneity (see below). Data were pooled on the same interventions (same route of administration and drug regimen), where appropriate and separate analyses for the intravenous and intramuscular control regimens were conducted. Adverse event data were presented in a table, a meta-analysis and in a narrative summary of the findings.

Heterogeneity was assessed by visually examining the forest plots (for overlapping confidence intervals and outliers) and using the chi-squared test for heterogeneity with a 10% level of statistical significance. Because statistically significant heterogeneity was detected for diarrhoea (an adverse event), the DerSimonian and Laird random-effects model was used to pool data for this outcome.

It was intended to use subgroup analyses or meta-regression to explore participant age (less than five years versus five years or older), disease severity (uncomplicated versus severe) and different galenic quinine formulations (solution, intrarectal cream or suppositories) as potential sources of heterogeneity, but this was not possible because of the uniformity of the age of participants (children less than 15 years of age only) and the small number of trials of people with severe disease and different galenic formulations. It was intended to investigate publication bias using funnel plots, but it was considered to be inappropriate in view of the small number of trials included.

## Results

### Description of studies

The search strategy identified 14 potentially relevant studies. Eight randomized controlled trials (involving 1,247 children) [20,24-30], one of which was quasi-randomized [24], fulfilled the inclusion criteria. The same principal investigator led seven of the trials. The trials recruited children up to 15 years of age who were hospital in-patients in Burkina Faso [26,30], Niger [20,24,25,27,29] or Togo [28]. The criteria for inclusion in the trial were: the degree of parasitaemia (>1,000 asexual *P. falciparum *stages/μl) [20,24-27], vomiting [20,24,26,28] and severe malaria [25,29]. All the trials had diarrhoea as an exclusion criterion; some also used treatment with antimalarial drugs before admission (seven trials), other documented causes of fever (six trials) and forms of severe malaria (five trials). Details on the number of participants, interventions and outcomes investigated are listed in Table 1. Five trials (227 children) compared intrarectal with intravenously administered quinine. Four trials (179 children) compared intrarectal quinine administered for two to three days with intravenous quinine administered for the same duration [20,24-26], and one trial compared single doses of intrarectal quinine and intravenous quinine that were followed by a three-day course of oral quinine [27]. Participants that had a two-day quinine course completed a total of five days of treatment with oral chloroquine [26] or quinine [27]. Six trials (1,122 children) compared intrarectal with intramuscular quinine. Four trials compared intrarectal and intramuscular quinine administered for three days [20,24,28,30], one trial did not mention the duration of treatment [29] and one trial gave a single dose of intrarectal or intramuscular quinine followed by three days of oral quinine [27].

**Table 1:** Trial participants, interventions and outcomes

**Reference**	**Participants**	Quinine: routes of administration and doses*	**Outcomes**
[20]	21 children aged 2 to 14 years in Niger	(1) Intrarectal (8 mg/kg; gluconate cream) (2) Intramuscular (4.7 mg/kg) (3) Intravenous (4.7 mg/kg) All 8 hourly for 3 days	Death, parasite clearance by day 7, adverse events
[24]	66 children aged 2 to 15 years in Niger	(1) Intrarectal (11.8 mg/kg; given as Quinimax^® ^diluted with water in a syringe) (2) Intravenous (7.4 mg/kg) (3) Intramuscular (7.4 mg/kg) All 12 hourly for 3 days	Death, parasite clearance time, parasite clearance by day 7, fever clearance time, adverse events
[25]	77 children aged 2 to 15 years in Niger	(1) Intrarectal (11.8 mg/kg once then 8.8 mg/kg 8 hourly for 2 days; Quinimax^® ^solution diluted in water via a syringe) (2) Intravenous (4.7 mg/kg 8 hourly for 2 days)	Death, parasite clearance time, fever clearance time, days in hospital, coma recovery time, time until drinking began, adverse events
[26]	48 children aged 2 to 15 years in Burkina Faso	(1) Intrarectal (bichlorhydrate diluted in a syringe) (2) Intrarectal (cinchona alkaloid diluted in a syringe) (3) Intravenous (bichlorhydrate) (4) Intravenous (cinchona alkaloid) All 8 mg/kg quinine 8 hourly for 2 days	Parasite clearance by 48 hours and 7 days
[27]	15 children aged 2 to 14 years in Niger	(1) Intravenous (4.74 mg/kg) (2) Intramuscular (4.74 mg/kg) (3) Intrarectal (11.85 mg/kg) All single dose	Death, parasite clearance by day 7, adverse events
[28]	64 children aged 0 to 15 years in Togo	(1) Intrarectal (15 mg/kg: Quinimax^® ^solution diluted in a syringe) (2) Intramuscular (12.5 mg/kg) Both 12 hourly for 3 days	Death, parasite clearance by day 2, adverse events
[29]	58 children aged 2 to 15 years in Niger	(1) Intrarectal (17.9 mg/kg once, then 11.75 mg/kg 12 hourly; Quinimax^® ^diluted in a syringe (2) Intramuscular (7.5 mg/kg 12 hourly)	Death, fever clearance time, duration of hospitalization, coma recovery time
[30]	898 children aged 1 to 15 in Burkina Faso	(1) Intrarectal (20 mg/kg; Quinimax^® ^diluted in a syringe) (2) Intramuscular (12.5 mg/kg) Both 12 hourly for 3 days	Death, fever clearance time, adverse events

*Quinine dose given as quinine base

### Methodological quality of included studies

Four trials did not describe the method used to generate the allocation sequence [20,26,28,30], three trials used random-numbers tables [25,27,29] and one trial used alternate allocation (quasi-randomization) [24]. None used procedures to conceal allocation. The nature of the interventions used did not allow blinding of participants and carers, and none of the outcome assessors were blinded. One trial excluded one participant (1.3%) from the analysis [25]. Another trial [24] could only analyse the parasite clearance time for 20 out of the 66 trial participants (30%) without providing a reason for the missing participants. The other six trials did not report on any exclusion or drop out of randomized participants. None of the trials analysed data on an intention-to-treat basis.

None of the trials reviewed contained a power calculation to determine the number of participants required to achieve sufficient statistical power for a clinically meaningful difference in effect of the different modes of administration to be detected.

### Outcomes

Each trial reported on at least one of the review's pre-specified outcomes, but none reported on the time lapsed before eating resumed. They also reported on other outcomes not analysed in this systematic review: time for parasitaemia to fall by 50% (three trials), percentage of initial parasitaemia after 24 hours (one trial) and 48 hours (three trials), time before one could sit (one trial); time before one could walk (one trial) and time for the body temperature to fall below 37.5°C (one trial).

### Effects of intrarectally versus intravenously administered quinine

**Figure 1:**
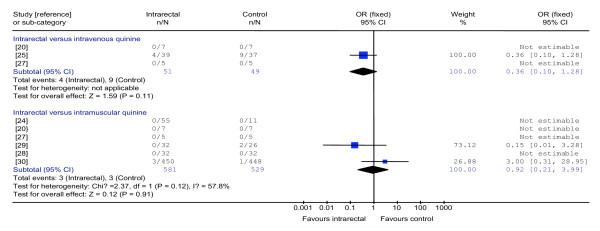
Meta-analysis of effects of intrarectal, intravenous and intramuscular quinine on death

**Figure 2:**
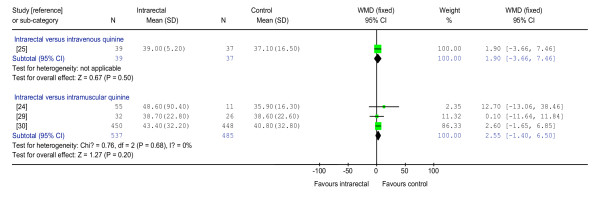
Meta-analysis of effects of intrarectal, intravenous and intramuscular quinine on fever clearance time (hours)

There was no statistically significant difference between the interventions for the number of deaths (100 participants, three trials) (Figure 1), parasite clearance at 48 hours (24 participants, one trial), parasite clearance time (76 participants, one trial), fever clearance time (76 participants, one trial) (Figure 2), duration of hospitalization (76 participants, one trial) coma recovery time (76 participants, one trial) and time until drinking began (76 participants, one trial). Participants in two trials were reported to have cleared their parasites by 48 hours [26,28] while four trials reported that all participants had cleared their parasites by day seven [20,21,24,26].

Two trials reported on adverse events and specifically mentioned the absence of rectal irritation and diarrhoea [25,26]. Barennes [26] also reported mucoid stools in four children in the intrarectal group.

### Effects of intrarectally versus intramuscularly administered quinine

There was no statistically significant difference between the interventions in the number of deaths (1,110 participants, six trials) (Figure 1), parasite clearance at 48 hours (64 participants, one trial), fever clearance time (1,022 participants, three trials) (Figure 2), duration of hospitalization (58 participants, one trial) and coma recovery time (58 participants, one trial). Four trials (154 participants) also reported that no deaths occurred. Parasite clearance time was statistically significantly longer in the participants treated with intrarectal quinine (mean 46.5 (standard deviation 22.0) hours) compared with those treated with intramuscular quinine (27.4 (9.5) hours) (WMD 19.10 hours, 5.20 to 33.00; 20 participants, one trial) [24]. However, all ninety participants in three trials [20,24,27] were reported to have cleared their parasites by day seven.

Data on adverse events were accessible for statistical analysis in three trials [23,27,29]. Assimadi [28] reported no statistically significant difference between painful swelling at the site of application (OR 0.13, 0.01 to 2.62, random-effects model) and pain at the site of application after administration (OR 0.1, 0.01 to 1.89, random-effects model). Barennes [30] reported that pain during administration occurred in four out of 450 participants given intrarectal quinine and 412 out of 448 participants given intramuscular quinine (OR 0.00, 0.00 to 0.00), with a test result for overall effect of Z = 13.46 (P < 0.00001). There was no statistically significant difference in the number of participants with mild diarrhoea between the groups (1,022 participants, three trials). The largest trial [30] also documented adverse events affecting stool consistency and content, pain in the rectum, effects on the rectal mucosa, as well as effects specific to intramuscular administration (Table 2).

**Table 2:** Descriptive adverse event data from Barennes [30]

**Adverse event**	**Intrarectal (number affected/total number of patients, %)**	**Intramuscular (number affected/total number of patients, %)**
Liquid stool (<3/day)	109/450 (24.2)	7/448 (1.5)
Mucoid stools	296/450 (65.7)	23/448 (5.10)
Blood in stool	20/450 (4.4)	3/448 (0.7)
Painful contractions	64/450 (14.2)	No data
Inflammation at the injection site	No data	358/448 (80)
Tenesmus	56/450 (12.4)	No data
Number investigated by anoscopy with a single microulceration healing within 24 hours	4/259 (1.5)	No data
Multiple microulcerations recovering by day 7	1/259 (0.4)	No data
Difficulty in walking	No data	67/448 (15)
Sciatic paresthesia	No data	1/448 (0.2)
Fever recurrence due to inflammation or infection of the injection site	No data	30/448 (6.6)

Three trials that reported on adverse events did not separate the results for the intrarectal, intramuscular and intravenous groups [20,24,27]. They specifically commented on the absence of rectal irritation (all three trials) and diarrhoea [20,27]. Barennes [20] also observed slight pain at the injection site in the intramuscular group.

## Discussion

Seven out of the eight trials that met the inclusion criteria consisted of less than 80 participants. This small number of participants increased the probability of missing a clinically important difference between groups. Only for the outcomes of death, fever clearance time and mild diarrhoea (an adverse event) were there two or more trials available for a meta-analysis. Only three of the trials documented the use of adequate randomization; adequate randomization was particularly important because blinding of participant and carer was not possible. This has increased the risk of a selection bias. All but one trial were conducted under the lead of one author, H. Barennes. The only pharmaceutical company producing an intrarectal quinine preparation, Quinimax^®^, sponsored four of the trials [24,25,29,30].

There was no statistically significant difference between intrarectal and parenteral quinine administration in terms of death, course of *P. falciparum *malaria or diarrhoea. Intrarectal administration also had the benefit of being less painful.

Parasite clearance time was longer in participants given quinine intrarectally as compared with intramuscular treatment in one trial [24], but it was not different when intrarectal administration was compared with intravenous administration in another trial [25]. This may have been because parasitaemia in the trial Barennes published in 1995 [24] was three times higher at baseline in the intrarectal group.

Statistically significant heterogeneity was observed when analysing the mild diarrhoea adverse event outcome. This may have been due to different definitions of diarrhoea, which was only clearly defined in one trial [30], and the large weight attributed in the meta-analysis to one small trial in which two out of five participants in the control group were affected [24]. Persistent pain at the injection site due to inflammation with the recurrence of fever seemed to be common with intramuscular injection and is an adverse effect not observed with the intrarectal route. This should be taken into consideration in the design of future trials comparing the two modes of administration. The occurrence of rectal mucosal ulcerations with intrarectal administration and its significance should also be assessed in all future trials. Adverse effects unique to the methods of intramuscular administration (sciatic nerve injury, infections with other viral and bacterial pathogens through contaminated needles) or intravenous injection (infections) are absent in intrarectal administration and cannot be addressed in a trial setting where administration is performed by trained personnel with adequate supply of consumables.

All trials included in the review had only children as participants, therefore, the results may not be applicable to adults.

Only two small randomized controlled trials (135 children) comparing intrarectal with intravenous [25] or intramuscular treatment [29] had participants with severe malaria. Limited data are, therefore, available on the effectiveness of intrarectal quinine in life-threatening forms of malaria.

## Conclusion

In a series of unconcealed trials from one research group, intrarectal administration appeared to be superior to intramuscular or intravenous administration in terms of reduced pain during administration. No difference in the effect on parasites and clinical illness was detected for the use of intrarectal quinine compared with other routes, but most trials were small. Thus, intrarectal application may be superior for uncomplicated falciparum malaria in children in cases in which administration of antimalarial drugs by mouth is not possible. There is insufficient evidence of the effectiveness of intrarectal quinine in severe falciparum malaria in children.

Further large-scale concealed randomized controlled trials are required to investigate intrarectally administered quinine in severe falciparum malaria in children and in all forms of falciparum malaria in adults. Further trials should focus on adverse effects including short-term and long-term effects on the rectal mucosa with intrarectal administration. Trials investigating the use of intrarectal quinine in the primary care setting, its role in preventing hospital admission and early treatment in the community, preventing complications associated with late presentation at healthcare facilities, are also desirable.

## Authors' contributions

Michael Eisenhut and Aika Omari developed the protocol, searched the literature and extracted data. Michael Eisenhut wrote the review, which Harriet G MacLehose critically revised. All authors read and approved the final manuscript.
